# Comparison of Treatment Outcomes among Sibling Oocytes Using Different Culture Systems—Conventional IVF versus INVOcell Device—And Evaluation of INVOcell User Satisfaction: The INVOcIVF Study

**DOI:** 10.3390/ijerph191912391

**Published:** 2022-09-29

**Authors:** Wan Syahirah Yang Mohsin, Nor Shaireen Abdullah Chue, Fazilah Abdul Hamid, Muhammad Azrai Abu, Sukhilmi Othman, Norazilah Mat Jin, Shu Yuan Woon, Abdul Kadir Abdul Karim, Mohd Faizal Ahmad

**Affiliations:** 1 Advanced Reproductive Centre HCTM Cheras, Kuala Lumpur 56000, Malaysia; 2 Department of Obstetrics & Gynecology, Hospital Tuanku Azizah (HTA), Kuala Lumpur 50300, Malaysia; 3 Hospital Bersalin Sukhilmi, No. 1-G, Jalan Coco Drive 3, Taman Bandar Senawang, Senawang 70450, Malaysia; 4 Reproductive Unit Faculty of Medicine, Universiti Teknologi MARA, Sg Buloh Campus, Kuala Selangor 45800, Malaysia; 5 Hospital Umum Sarawak, Kuching 93586, Malaysia

**Keywords:** in vitro fertilization, intravaginal culture, INVOcell, blastulation, fertilization, user satisfaction, quality of life

## Abstract

INVOcell is considered an alternative to conventional IVF proposed for intravaginal embryo culture; however, implementation is still low because evidence is scanty regarding its outcome and, most importantly, the device’s user satisfaction. Thus, we aim to compare the embryo outcome of sibling oocytes following INVOcell culture with conventional IVF (cIVF) by assessing its clinical outcome (fertilization, blastulation rate, and good embryo quality) and the user satisfaction evaluation based on a local validation questionnaire. A prospective study was done at a university-setting hospital for 12 months (July 2021–2022). The oocytes collected were divided into INVOcell and cIVF groups equally. Inclusion criteria included <40 years old and body mass index (BMI) < 30 kg/m^2^. The pre- and post-satisfaction questionnaires were assessed. In total, 23 women were included following standard controlled ovarian stimulation (COS). The mean age was 32.9, and the mean BMI was 24.9 kg/m^2^. Most of them suffered from tubal factors. A total of 252 oocytes were collected and incubated accordingly (cIVF; 138, INVOcell; 114). The blastulation rate was superior in the INVOcell group (*p* = 0.16); otherwise, the fertilization rate and good embryo quality were not significantly different between both methods (*p* > 0.05). Overall, women were satisfied with the INVOcell device as they were adequately advised, follow-up was scheduled, and the lowest score was obtained for all side effects of the device. Although both methods produce similar fertilization rates and good-quality embryos, the blastulation rates were better in the INVOcell group. Functionally, it is a user-friendly device and tolerable. Therefore, INVOcell can be used as an alternative method for reproductive treatment in carefully selected patients without jeopardizing the IVF outcomes.

## 1. Introduction

Intravaginal culture (IVC) was proposed by Ranoux et al. in 1988 to reduce the overall burden of setting up an embryological laboratory and to facilitate access to reproductive care in low-resource settings [1]. The idea evolved over years and led to the development of INVOcell^®^. The IVC technique using INVOcell^®^ involves a “small vaginal tube” serving as a gas-permeable device to culture oocytes and sperm after extraction [2]. The device is inserted into the vaginal cavity, acting as a “natural incubator” for fertilization and embryo development. The formed embryo is then examined and utilized in the in vitro fertilization (IVF) cycle [3]. As the fertility rate is declining worldwide, the requirement for fertility treatment, mainly IVF, is increasing. However, establishing IVF centers is expensive; thus, most centers are confined to urban areas where the patients can afford the treatment [4,5]. In addition, the cost includes training highly-skilled personnel coupled with incubator systems. Therefore, the uptake of fertility treatment is low despite the high demand. Development of a low-cost procedure can be an alternative to overcome this problem [6]. To date, our center was selected as the first in Malaysia to initiate the use of INVOcell^®^ as a low-cost IVF procedure [7]. However, most women avoid the use of any vaginal device because of discomfort, pain, or infection; thus, we coupled the aim of our study to assess the overall outcome and satisfaction of using INVOcell^®^ to ensure acceptability of this alternative technique among women.

## 2. Materials and Methods

### 2.1. Study Design

**Figure 1:**
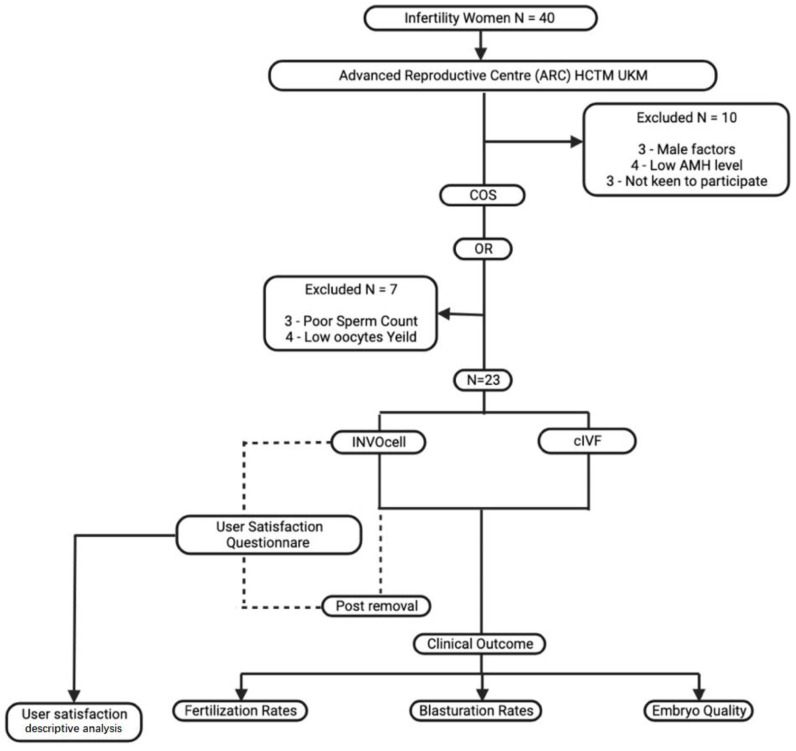
Flow of the study.

This is a prospective study on selected patients who underwent IVF treatment at the Advanced Reproductive Centre (ARC), at Hospital Canselor Tuanku Muhriz HCTM, at the National University of Malaysia, from January 2021 to January 2022. This study was approved by the Human UKM Research Ethics Committee (JEP-2020-724). Women aged 18–39 years with subfertility, defined by the presence of at least more than six oocytes retrieved during oocyte retrieval (OR), were recruited and underwent IVF using INVOcell and conventional IVF (cIVF). Women with poor ovarian reserve (less than 1 ng/mL), low oocytes yield (fewer than six) during oocytes retrieval (OR), two or more IVF failures, and severe male factors during recruitment or at the day of OR were excluded (Figure 1).

### 2.2. Study Protocol

**Figure 2:**
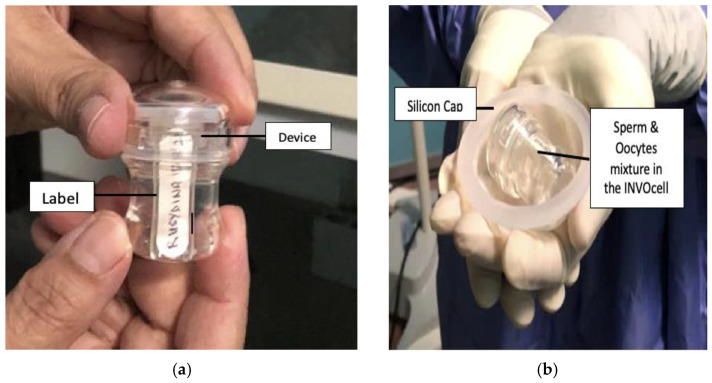
Preparation of the device for INVOcell procedure. (**a**) The device is labelled with the woman’s registration number and name. (**b**) The device is ready for vaginal insertion.

Controlled ovarian stimulation (COS) was performed following the standard ARC protocol. In brief, all women received urofollitropin (Folliculin^®^) and human menopausal gonadotrophin (Humog^®^) in an antagonist cycle (Cetrotide^®^). Follicular tracking was conducted accordingly on days 7th and 8th of the cycle. Subsequently, the urinary hCG (Hucog^®^) was given as a trigger when two or more follicles were at least 18 mm^3^. The OR was performed 36 h following the trigger. Following the OR, half of the total oocytes from the same patient were placed into the INVOcell device, and the other half were cultured following the cIVF protocol. The INVOcell device was prepared following a previously described protocol [8]. First, the semen was prepared using the “swim-up” technique with at least 1 million spermatozoa loaded into the device. The device was labelled and, subsequently, was placed inside the woman’s vagina for at least 5 days (Figure 2). Then, the fertilization, blastocyst, and embryo quality were assessed, and the embryo transfer was performed if suitable or frozen for future use. 

### 2.3. Evaluation of INVOcell Device 

The couple was seen before OR for counseling on the INVOcell device and procedure. Consent was taken, and continuation of care was given following the INVOcell insertion. All possible side effects of the device were explained accordingly. After removing the device, the locally validated questionnaire (a doctor-administered questionnaire) was given, assessing the overall women’s views, experiences, and satisfactions following the treatment. Ten questions were evaluated: information assessment before insertion, side effects following insertion of the device, follow-up information after the device was inserted, and overall expectation and future use or recommendation. All of these questions were answered as “Yes”, “No”, or “Unsure.” Descriptive analysis was used from the frequency and percentage of the answers. 

### 2.4. Fertilization, Blastulation, and Embryo Quality Evaluation

Oocyte maturation was assessed accordingly via microscopy to confirm the presence of a polar body (meiosis II). As the INVOcell group was not evaluated for two pronuclei (2PN) after 16–18 h, the fertilization and blastulation rates were obtained by comparing the percentage of blastulation rates at day five in both groups. In the INVOcell group, the fertilization rates were determined by the number of oocytes fertilized and the number of oocytes placed in the device. Otherwise, the blastulation rates were determined by the number of blastocyst formations relative to the number of oocytes fertilized. In the cIVF group, PN check was performed following 16–18 h culture, and the fertilization rates were calculated. The embryos were cultured following the local protocol: they were placed under individual mineral oil in 10 µL droplets of Omni Medium (Vitrolife^®^) supplemented with 10% human albumin serum and cultured up to the day five blastocyst stage. Following day five culture for both groups, the inner cell mass and trophectoderm were assessed. Following Gardner scoring, type A considers compact, with many cells present; in type B, the cells are loosely grouped. The tight epithelial network was considered type A for the trophectoderm, and loose network cells were labeled as type B. Both groups graded good embryo quality, at most minuscule 4 BB and above.

### 2.5. Statistical Analysis

Statistical analysis was conducted using SPSS version 28 (IBM, Armonk, NY, USA). Descriptive data are expressed as mean ± SD (standard deviation) or frequencies (no. of cases) and percentages when appropriate. Comparison between groups was performed using the Chi-square test and student’s *t*-test, depending on the data distribution. Statistical significance was considered at *p* < 0.05.

## 3. Results

**Figure 3:**
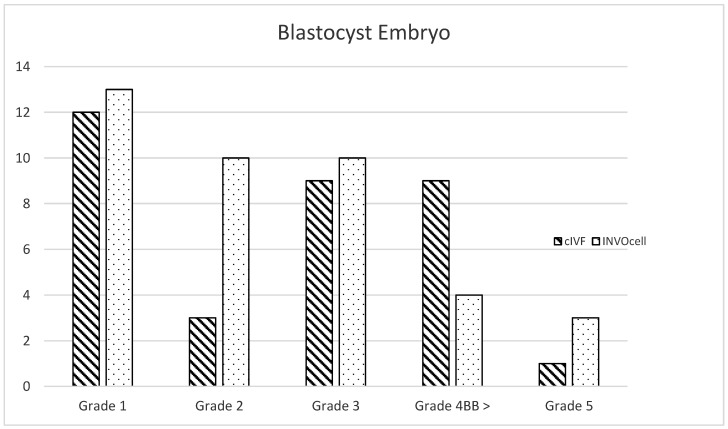
Grade of Blastocyst embryos distributed by Gardner grading scoring for INVOcell^®^ and cIVF.

**Figure 4:**
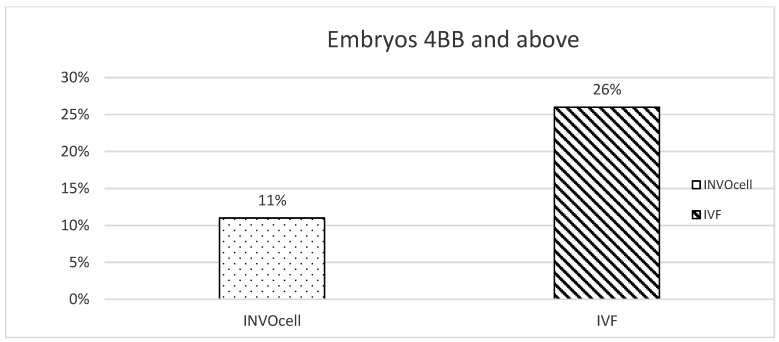
The number of good-quality embryos 4BB and above for IVC and IVF.

About 40 women were assessed. At least 10 were excluded during recruitment due to male factors, low AMH level, or not being keen to participate. Subsequently, 30 women underwent COS as described earlier and proceeded with OR. However, three were excluded due to unexpectedly poor sperm count, in which intracytoplasmic sperm injection (ICSI) is required. The other four women yielded less than six oocytes, thus not fulfilling the inclusion criteria. (Figure 1). Thus, a total of 23 women with a mean age of 32.9 and a mean BMI of 24.9 kg/m^2^ were included in our study. Most of the participants suffered from tubal factors (39.1%), and some had endometriosis (13.1%; Table 1). Twenty-three COS cycles were performed, and 252 oocytes were collected. All the cycles underwent retrieval and yielded at least four or more oocytes. Approximately 115 oocytes were cultured via cIVF, whereas 138 oocytes were placed in INVOcell^®^ (Table 2). At least one oocyte was fertilized following INVOcell^®^ incubation and three oocytes following cIVF treatment. Otherwise, no statistical difference existed between the two culture systems (*p* = 0.331). Otherwise, 33.9% (39/115) from cIVF and 32.6% (45/138) from INVOcell^®^ oocytes were cultured up to the blastocyst stage (Table 2). Our study revealed that the blastulation rate was significantly higher in the INVOcell^®^ group than in the cIVF group (*p* = 0.016; Table 3). However, the blastocysts were lower in good-quality scoring (Figure 3). In addition, 11% (5/45) of the INVOcell^®^ blastocysts were graded equal to or greater than 4BB, whereas 26% (10/39) were observed from the cIVF group (Figure 4). Nevertheless, no significant difference was found (*p* = 0.636; Table 3). Most women reported satisfaction with the INVOcell device during the advised and scheduled subsequent follow-up. Most of them scored low for side effects of the device and concurred that the treatment met their expectations. They were also likely to choose the device again in the future and recommend it to their peers (Table 4).

## 4. Discussion

The fertility rate is declining worldwide. In Asian countries, the total fertility rate (TFR) dropped from 5.5 in the 1970s to at least 1.9 in 2021 [9]. This is considered alarming because it is below the replacement rate of 2.1 [10]. Although conceiving later in life has become a modern trend, fertility problems remain a significant contributor to this outcome; therefore, reproductive treatment is still considered vital in alleviating these matters. The reproductive industry and related technologies have advanced tremendously. However, such techniques are expensive and mostly limited to large cities [5,11]. Therefore, a simple yet cost-effective method must be developed to ensure that reproductive treatment can be advocated similarly in smaller district areas [11]. IVF is not a new technique. Since the successful delivery of Louis Brown on 25 July 1978, more than eight million babies have already been born successfully through the same process worldwide [12]. The cIVF practice progressed according to new techniques and scientific research to improve the outcomes and achieve more live births. The intracytoplasmic sperm injection (ICSI) was introduced in the 1990s as one of the most dramatic breakthroughs in assisted reproductive technology (ART), initially for male-factor subfertility [13]. To date, ICSI use has expanded from 15.4% to 66.9% from 1996 to 2012 in non-male-factor cases [14]. Although ICSI may increase the likelihood of fertilization, the general use of ICSI for all cases of infertility is not recommended in ART [15,16]. Possible oocyte damage is one of the potential problems with this invasive technique, which is unpredictable and unsystematic; therefore, most centers still selectively utilize ICSI. In the ICSI case for non-male factors, the fertilization rate of the IVF group was significantly better than that of the ICSI group [16].

Therefore, in addressing the issue of complex techniques using ICSI and expensive incubators concerning modern IVF procedures, most districts cannot provide this service because of a lack of financial support [17,18]. Due to the increasing demand for reproductive treatment, IVF, and these limitations, the intravaginal culture system has been developed to provide a simple way of incubating embryos and facilitating IVF treatment in rural areas. In this technique, the sperm and oocytes are cultured in the gas-permeable device (Figure 4). This INVOcell device is placed inside the vagina cavity, which then acts as a natural incubator providing a stable pressure of CO^2^, O^2^ level, and body temperature to facilitate fertilization and embryo development [19]. However, the embryos are sensitive to in vitro manipulation and changes to the culture environment. Therefore, the ability of the vagina to sustain a proper microenvironment influences the formation of good early-stage embryos and the subsequent blastulation rate [20]. Our study was in concert with these findings. In the INVOcell culture system, the vagina serves as a natural incubator, which helps maintain temperature and provides a low oxygen and high CO^2^ environment [8,21]. This condition provides an advantage in eliminating potential fluctuation in those parameters with the conventional IVF method, which may negatively affect embryo development [21]. Nevertheless, the fertilization rates were reported to be comparable for both methods, similar to our findings [1,22,23,24,25]. A reduction of the observation frequency of embryos outside the incubator can enhance embryo quality and blastocyst formation [20]. Total blastocyst formation rate, the proportion of good-quality blastocysts, and the number of cryopreserved blastocysts per cycle were significantly lower than embryo monitoring daily, as observed with the conventional method [26]. A better outcome was obtained using time-lapse embryo monitoring, which is expensive and limited to prominent centers [27]. Conversely, the assessment of 2PN and regular fertilization for conventional IVF and ICSI is feasible within 17 + 1 h following insemination. Unlike the INVOcell system, fertilization assessment can only be performed on day five following device removal. However, although the normality of PN assessment is not attainable, the outcome is not inferior to the conventional method. The abnormal PN embryo is usually arrested at day five or rarely it forms a good blastocyst, which can be transferred [28]. Additional tests such as pre-genetic testing can be offered prior to transfer or non-invasive prenatal testing as an adjunct assessment for the fetus to ensure normality. Otherwise, the fertilization rate assessment for our INVOcell system arm was based on the number of fertilized oocytes to form a blastocyst. Thus, no difference in fertilization rate can be found between INVOcell and conventional IVF, which agrees with previous reports [8,29].

Few studies compared the blastocyst formation rates between INVOcell and conventional IVF [29,30]. However, a recent study has reported that INVOcell results in a better blastocyst formation rate than cIVF [30,31,32]. This evidence is consistent with our findings. As established, the INVOcell culture gives an advantage to a stable environment that promotes optimum blastocyst development mainly due to the ability to maintain the fluctuating factor compared to cIVF which generally offers a static environment for blastocyst culture, theoretically leading to less optimization of the dividing cells [1,8,24]. Therefore, some centers adopt the “shaker” incubator to mimic the natural cycle [33]. Otherwise, INVOcell and conventional IVF show no difference in the producibility of good-quality embryos. Other studies had the same conclusion [3,8,22,30,31].

In addition to clinical outcomes, our study offered the experiences of real women who used the INVOcell device. Scanty evidence had been reported regarding the experience of device users, although it is vital in implementing this method [30]. Most women require an excellent explanation of the system, and proper follow-up should be offered after insertion. Our study also consolidates information about the side effects, mainly vaginal discomfort and discharge, experienced by these women. Considering the INVOcell device as a foreign body, it does irritate the normal flora and forms a discharge [34]. However, it does not cause ascending infection because the discharge is mainly inflammatory rather than being an infection. Surprisingly, most women can tolerate the discharge and alleviate it daily with a sanitary pad. Nevertheless, the device and protection cap can also lead to abrasion or laceration in the thin vaginal wall. Fortunately, such effects were not observed in our study. The device size can also provoke anxiety prior to insertion. However, following insertion, few women experienced discomfort and pain, and denied any adverse side effects. The fixed and comfortable silicon cap plays an essential role in facilitating daily movement and activity, such as urination and defecation, without the device accidentally falling out. This led to a great satisfaction among the users, and the device’s possible falling out was considered the ultimate “nightmare” for all the users. Most of the users agreed that the device experience met the expectation as counselled by the clinician. Otherwise, the removal of INVOcell was considered accessible, and none of them experienced discomfort, pain, or trauma. Thus, most of the users were willing to repeat the treatment if needed and recommend it to their friends or family members. Taking these experiences as a benchmark, we now consolidate the notion that our clinical experiences concur with established shreds of evidence and couple it with the experiences of our study’s participants. Based on the findings of this study, we recommend INVOcell as an alternative to cIVF. With this device, we hope that fertility treatment can be offered widely at no additional cost to improve the overall TFR worldwide.

### Strengths and Limitations of This Study

To our knowledge, this study is the first to consolidate clinical outcome with user satisfaction in Asia. Although there are publications covering intravaginal culture, such publications do not integrate the value of intravaginal culture with user satisfaction. Therefore, our study will be a landmark reference in this regard. Our study also has a limitation. The gathering of information is considered limited because only one center’s data were obtained. This is because we are the pioneer of intravaginal culture in Malaysia. Our data are, therefore, limited to the scope of what we can achieve. Otherwise, in Asia, intravaginal culture is considered new and this fact, coupled with social taboos relating to culture and religion, may affect the implementation of this technique.

## 5. Conclusions

Although cIVF improves the overall reproductive outcome, it is costly and not applicable in most rural and district areas, therefore, the INVOcell can be utilized as a cost-effective alternative to cIVF without sacrificing comfort and outcome.

## Figures and Tables

**Table 1:** Classification of women participating in the study on the basis of age- and BMI-related profiles and infertility factors.

Age	32.9 years old
BMI	24.9 kg/m^2^
Fertility Factors	*n* (%)
○ Endometriosis	9 (39.1)
○ Tubal Factor	3 (13.1)
○ Adenomyosis	5 (21.7)
○ PCOS	6 (26.1)

**Table 2:** Developmental potential of IVF-derived embryos according to the culture system.

Type of Intervention	cIVF	INVOcell^®^
No. of oocytes	*n* (mean) 115 (4.987)	*n* (mean) 138 (6)
No. of blastocysts	*n* (%) 39 (33.9)	*n* (%) 45(32.6)

**Table 3:** Overall treatment outcomes in both groups.

**Fertilization Rates**			
INVOcell^®^ (*n* = 23) Median (IQR)	cIVF (*n* = 23) Median (IQR)	Z statistic	*p*-value ^a^
1.0 (0.2–4.0)	3.0 (1.0–4.0)	−0.973	0.331
**Blastulation Rates**			
INVOcell^®^ (*n* = 23) Median (IQR)	cIVF (*n* = 23) Median (IQR)	Z statistic	*p*-value ^a^
1.0 (0.2–1.0)	0.5 (0–1.0)	−2.420	0.016 *
**Good-Quality Embryos**			
INVOcell^®^ (*n* = 23) Median (IQR)	cIVF (*n* = 23) Median (IQR)	Z statistic	*p*-value ^a^
0 (0–0.33)	0 (0–0.25)	−0.473	0.636

^a^ Wilcoxon signed-rank test; * statically significant.

**Table 4:** INVOcell Users Evaluation Outcomes.

Q1	**Did you have all information and advice that you needed before insertion of the device?**		
	Answers	Frequency	Percentage (%)
	No	0	0
	Unsure	0	0
	Yes	23	100
Q2	**Did you experience an increase in vaginal discharge?**		
	Answers	Frequency	Percentage (%)
	No	12	52.2
	Unsure	0	0
	Yes	11	47.8
Q3	**Did you experience per-vaginal bleeding?**		
	Answers	Frequency	Percentage (%)
	No	22	95.7
	Unsure	0	0
	Yes	1	4.3
Q4	**Did you have any other side effects following intravaginal culture device insertion?**		
	Answers	Frequency	Percentage (%)
	No	23	100
	Unsure	0	0
	Yes	0	0
Q5	**Did the intravaginal culture device affect your daily life?**		
	Answers	Frequency	Percentage (%)
	No	23	100
	Unsure	0	0
	Yes	0	0
Q6	**Did you have any discomfort following intravaginal culture device insertion?**		
	Answers	Frequency	Percentage (%)
	No	23	100
	Unsure	0	0
	Yes	0	0
Q7	**Did you think that the follow-up you received was adequate?**		
	Answers	Frequency	Percentage (%)
	No	12	52.2
	Unsure	0	0
	Yes	11	47.8
Q8	**Did the treatment meet your expectations?**		
	Answers	Frequency	Percentage (%)
	No	0	0
	Unsure	5	21.7
	Yes	18	78.3
Q9	**Was the removal of the device painful?**		
	Answers	Frequency	Percentage (%)
	No	23	100
	Unsure	0	0
	Yes	0	0
Q10	**In a similar situation would you chose the intravaginal culture device again or recommend it to a good friend?**		
	Answers	Frequency	Percentage (%)
	No	0	0
	Unsure	5	21.7
	Yes	18	78.3

## Data Availability

Not applicable.

## References

[1] Ranoux C., Aubriot F.X., Dubuisson J.B., Cardone V., Foulot H., Poirot C., Chevallier O. (1988). A new in vitro fertilization technique: Intravaginal culture. Fertil. Steril..

[2] Fukuda M., Fukuda K., Ranoux C. (1996). Unexpected low oxygen tension of intravaginal culture. Hum. Reprod..

[3] Frydman R., Ranoux C. (2008). INVO: A simple, low cost effective assisted reproductive technology. Hum. Reprod..

[4] Ombelet W. (2011). Global access to infertility care in developing countries: A case of human rights, equity and social justice. Facts Views Vis. Obgyn..

[5] Ombelet W., Cooke I., Dyer S., Serour G., Devroey P. (2008). Infertility and the provision of infertility medical services in developing countries. Hum. Reprod. Update.

[6] Gameiro S., Boivin J., Peronace L., Verhaak C.M. (2012). Why do patients discontinue fertility treatment? A systematic review of reasons and predictors of discontinuation in fertility treatment. Hum. Reprod. Update.

[7] Edition B.A. (2022). INVOcell Launched in Malaysia by Advanced Reproductive Centre, Hospital Canselor Tuanku Muhriz. https://www.prnewswire.com/news-releases/invocell-launched-in-malaysia-by-advanced-reproductive-centre-hospital-canselor-tuanku-muhriz-301612139.html.

[8] Lucena E., Saa A.M., Navarro D.E., Pulido C., Lombana O., Moran A. (2012). INVO procedure: Minimally invasive IVF as an alternative treatment option for infertile couples. Sci. World J..

[9] Post T.A. (2020). Declining fertility rates in ASEAN. Asian Post.

[10] Craig J. (1994). Replacement level fertility and future population growth. Popul. Trends.

[11] Katz P., Showstack J., Smith J.F., Nachtigall R.D., Millstein S.G., Wing H., Eisenberg M.L., Pasch L.A., Croughan M.S., Adler N. (2011). Costs of infertility treatment: Results from an 18-month prospective cohort study. Fertil. Steril..

[12] Dow K. (2019). Looking into the Test Tube: The Birth of IVF on British Television. Med. Hist..

[13] Morishita N., Ochi M., Horiuchi T. (2021). History and prospects of intracytoplasmic sperm injection (ICSI) and the development of golden hamster ICSI embryos. Reprod. Med. Biol..

[14] Glenn T.L., Kotlyar A.M., Seifer D.B. (2021). The Impact of Intracytoplasmic Sperm Injection in Non-Male Factor Infertility-A Critical Review. J. Clin. Med..

[15] Butts S.F., Owen C., Mainigi M., Senapati S., Seifer D.B., Dokras A. (2014). Assisted hatching and intracytoplasmic sperm injection are not associated with improved outcomes in assisted reproduction cycles for diminished ovarian reserve: An analysis of cycles in the United States from 2004 to 2011. Fertil. Steril..

[16] The Practice Committees of the American Society for Reproductive Medicine, The Society for Assisted Reproductive Technology (2020). Intracytoplasmic sperm injection (ICSI) for non-male factor indications: A committee opinion. Fertil. Steril..

[17] McPherson N.O., Vincent A.D., Pacella-Ince L., Tremellen K. (2021). Comparison of in vitro fertilisation/intracytoplasmic sperm injection on live birth rates in couples with non-male factor infertility and advanced maternal age. J. Assist. Reprod. Genet..

[18] Zagadailov P., Seifer D.B., Shan H., Zarek S.M., Hsu A.L. (2020). Do state insurance mandates alter ICSI utilization?. Reprod. Biol. Endocrinol..

[19] Swain J.E. (2010). Optimizing the culture environment in the IVF laboratory: Impact of pH and buffer capacity on gamete and embryo quality. Reprod. Biomed. Online.

[20] Zhang J.Q., Li X.L., Peng Y., Guo X., Heng B.C., Tong G.Q. (2010). Reduction in exposure of human embryos outside the incubator enhances embryo quality and blastulation rate. Reprod. Biomed. Online.

[21] Gardner D.K., Lane M., Calderon I., Leeton J. (1996). Environment of the preimplantation human embryo in vivo: Metabolite analysis of oviduct and uterine fluids and metabolism of cumulus cells. Fertil. Steril..

[22] Bonaventura L., Ahlering P., Morris R., Mouchel J., Scheiber M., Batzofin J. (2006). P-93: The INVOcell, a new medical device for intra vaginal fertilization and culture. Fertil. Steril..

[23] Ranoux C., Seibel M.M. (1990). New techniques in fertilization: Intravaginal culture and microvolume straw. J. Vitr. Fert. Embryo. Transf..

[24] Sterzik K., Rosenbusch B., Sasse V., Wolf A., Beier H.M., Lauritzen C. (1989). A new variation of in-vitro fertilization: Intravaginal culture of human oocytes and cleavage stages. Hum. Reprod..

[25] Taymor M.L., Ranoux C.J., Gross G.L. (1992). Natural oocyte retrieval with intravaginal fertilization: A simplified approach to in vitro fertilization. Obs. Gynecol..

[26] Krasnopolskaya K.V., Beketova A.N., Sesina N.I., Chinchenko N.K., Badalyan G.V., Sudarikova N.M., Bocharova T.V., Zakharchenko E.O. (2019). The effect of short-term disturbance of day 3 embryo culture on the development and implantation. Gynecol. Endocrinol..

[27] Kirkegaard K., Agerholm I.E., Ingerslev H.J. (2012). Time-lapse monitoring as a tool for clinical embryo assessment. Hum. Reprod..

[28] Chen X., Shi S., Mao J., Zou L., Yu K. (2020). Developmental Potential of Abnormally Fertilized Oocytes and the Associated Clinical Outcomes. Front. Physiol..

[29] Doody K.J., Broome E.J., Doody K.M. (2016). Comparing blastocyst quality and live birth rates of intravaginal culture using INVOcell™ to traditional in vitro incubation in a randomized open-label prospective controlled trial. J. Assist. Reprod. Genet..

[30] Jellerette-Nolan T., Cooper A.R., Doody K.J., Nichols J.E., Park J.K., Poe-Zeigler R.L., Khair A.F., Stong L.M., Paulson R.J., Daftary G.S. (2021). Real-world experience with intravaginal culture using INVOCELL: An alternative model for infertility treatment. FS Rep..

[31] Babayev E., Jain T. (2021). Intravaginal culture using INVOCELL: Is it a viable treatment option for infertility?. FS Rep..

[32] INVO Bioscience INVO Bioscience Announces Four Individual Poster Abstracts Discussing the Benefits of INVOcell. Proceedings of the 2021 American Society for Reproductive Medicine (ASRM) Congress & Expo.

[33] Evans C.W., Renee H. (2009). Effect of Angle of Turning and Shaking Agitation during Incubation on Embryo Development and Hatchability. https://repository.lib.ncsu.edu/handle/1840.16/133.

[34] Paterek J.A.E. (2022). Vaginal Foreign Body Evaluation and Treatment.

